# Multiscale-Engineered Muscle Constructs: PEG Hydrogel Micro-Patterning on an Electrospun PCL Mat Functionalized with Gold Nanoparticles

**DOI:** 10.3390/ijms23010260

**Published:** 2021-12-27

**Authors:** Megane Beldjilali-Labro, Rachid Jellali, Alexander David Brown, Alejandro Garcia Garcia, Augustin Lerebours, Erwann Guenin, Fahmi Bedoui, Murielle Dufresne, Claire Stewart, Jean-François Grosset, Cécile Legallais

**Affiliations:** 1 Centre de Recherche de Royallieu, Biomechanics & Bioengineering Laboratory, CNRS, Université de Technologie de Compiègne, 60203 Compiegne, France; megane.beldjilali-labro@utc.fr (M.B.-L.); rachid.jellali@utc.fr (R.J.); alejandro.garcia-garcia@utc.fr (A.G.G.); augustin.lerebours@utc.fr (A.L.); murielle.dufresne@utc.fr (M.D.); jean-francois.grosset@utc.fr (J.-F.G.); 2 Stem Cells, Ageing and Molecular Physiology Unit (SCAMP), Research Institute for Sport & Exercise Sciences (RISES), Liverpool John Moores University, Liverpool L3 5UX, UK; A.D.Brown@ljmu.ac.uk (A.D.B.); C.E.Stewart@ljmu.ac.uk (C.S.); 3 Centre de Recherche de Royallieu, Roberval Laboratory for Mechanics, CNRS, Université de Technologie de Compiègne, 60203 Compiegne, France; fahmi.bedoui@utc.fr; 4 Centre de Recherche Royallieu, TIMR (Integrated Transformations of Renewable Matter), ESCOM, Université de Technologie de Compiègne, 60203 Compiegne, France; erwann.guenin@utc.fr

**Keywords:** tissue engineering, scaffold, muscle, organoid, electrospinning, myotube

## Abstract

The development of new, viable, and functional engineered tissue is a complex and challenging task. Skeletal muscle constructs have specific requirements as cells are sensitive to the stiffness, geometry of the materials, and biological micro-environment. The aim of this study was thus to design and characterize a multi-scale scaffold and to evaluate it regarding the differentiation process of C2C12 skeletal myoblasts. The significance of the work lies in the microfabrication of lines of polyethylene glycol, on poly(ε-caprolactone) nanofiber sheets obtained using the electrospinning process, coated or not with gold nanoparticles to act as a potential substrate for electrical stimulation. The differentiation of C2C12 cells was studied over a period of seven days and quantified through both expression of specific genes, and analysis of the myotubes’ alignment and length using confocal microscopy. We demonstrated that our multiscale bio-construct presented tunable mechanical properties and supported the different stages skeletal muscle, as well as improving the parallel orientation of the myotubes with a variation of less than 15°. These scaffolds showed the ability of sustained myogenic differentiation by enhancing the organization of reconstructed skeletal muscle. Moreover, they may be suitable for applications in mechanical and electrical stimulation to mimic the muscle’s physiological functions.

## 1. Introduction

Reconstructing lost function or mass of skeletal muscle, caused by chronic diseases and traumatic injuries, is often difficult to achieve despite the highly regenerative nature of this tissue [1,2]. Natural, synthetic, or biohybrid biomaterials have been developed to replace or repair the structure of skeletal muscle. In addition, these constructs can represent suitable “native-like” tissue models for in vitro investigations, for better understanding of the muscle regeneration process, drug screening, the evaluation of physical protocols for the treatment of muscular diseases or injuries. Yet, none of them has successfully reinstated the structure/function of the native tissue. The highly organized structure of skeletal muscle in long parallel conductive fibers is undoubtedly a challenge for its reconstruction [3,4]. Controlling tissue organization in vitro by aligning myoblasts in preparation for the formation of myotubes is thus recognized as a crucial step [5]. An effective scaffold for skeletal muscle tissue engineering needs to provide such fundamental elements, i.e., an appropriate microenvironment that will allow muscle cells to grow, differentiate, and align to support the transmission of muscular force [6,7]. In recent years, several strategies have emerged to develop such biohybrid constructs, from the design of complex architecture to the use of external stimuli to foster the formation of parallel-aligned myofibrils. Some studies focused on the impact of the size (nano or micro) and topograhical design on myoblast alignment, and of fusion on the biomaterial. [8,9].

Nano topography-guided approaches was achieved by patterning parallel nanogroove on a polydimethylsiloxane (PDMS) substrates. It was shown to promote tissue differentiation and activate specific function [10,11]. The electrospinning technique [11,12] has also been widely used, as it provides fibers that mirror the structure of native collagen fibrils of extracellular matrix [13,14]. Yet, fibers must be highly aligned to offer cells a parallel support to form myotubes, otherwise cells follow the random organization of the electrospun fibers [15]. Photolithography, hot embossing, and soft lithography are helpful for creating micro-topographical cues. A previous study has shown enhanced alignment of myotubes using photolithography patterned micro-channels, spaced from 5 to 100 µm apart and with varying depths [16]. Meanwhile, other studies have investigated the effect of geometrical patterns, such as wavy, square, circle or plots, on myogenic differentiation [17,18]. They established that myotubes formed on large patterns were able to follow geometric cues in the microenvironment. Yet, the limitation of geometric guidance appeared on patterns with sharp corners and small curve radii. Although substrates from nano- to micro-features are acknowledged for favoring myotube formation, they have demonstrated some limitations as guidance cues when cell–substrate interaction is overridden by cell–cell interactions [17]. Therefore, some composite materials were created by combining various techniques, such as electrospinning and CoreShell, allowing the formation of nanofiber yarn embedded in hydrogel [19,20]. Finally, other approaches involving electrically conductive materials or polymers, such as carbon, gold, iron nanoparticles, polyaniline, and polypyrrole, have been investigated to mimic the natural environment and have shown they can improve the formation, maturation and guidance of myotubes [21,22,23,24].

Thus, it can also be hypothesized that coupling microtopography and electrospun nanofibers may closely mimic the topographical aspect of natural muscle bundles and efficiently enable the formation of aligned myotubes over large areas. Very recently, melt electrospinning writing (MEW) was used to fabricate 3D patterned microgrooves spaced by 100 to 300 µm on top of aligned poly(ε-caprolactone) (PCL) meshes both with/without gold coating. Spacing of 200 µm was found optimal for promoting myotubes formation [25]. The aim of the present study is thus to associate the effect of multiscale scaffolds and conductive nanoparticles on the preparation of nano-to-macro hierarchically organized tissue engineered skeletal muscle. One of the challenges is to work with a wider micropattern spacing than what is commonly used, in order to allow the formation of myofibrils or myofibers of physiological size. The easy-to-handle construct was integrated into this design phase to obtain in fine a biohybrid construct capable of sustaining external stimuli such as stretching cycles or electric pulse. We first manufactured a double-scaled scaffold, based on poly(ε-caprolactone) (PCL) electrospun nanofibers coated with gold nanoparticles (Au NPs) and then micropatterned it with polyethylene glycol (PEG) hydrogel lines. The PCL nanoscale fibers were expected to promote cell adhesion and guidance, while the gold particles were selected because of their nanostructure, easy surface functionalization process, and conductive properties. Finally, the PEG linear patterns were used to contribute to enhanced cell guidance. C2C12 myoblasts were then cultured on the scaffolds designed to evaluate their growth and differentiation into a new type of tissue engineered skeletal muscle.

## 2. Results

The surface area of the constructs (manufacturing process illustrated in Figure 9) was 6 cm^2^, for an average thickness of 91 µm ± 13 µm (*n* = 9). This manufacturing process led to four different types of scaffold: (I) electrospun poly-ε-caprolactone (PCL), (II) the same coated with gold nanoparticles (PCL-Au), (III) electrospun PCL with patterning of PEG hydrogel lines (PCL-PEG), and finally (IV) an electrospun PCL coated with gold nanoparticles and patterned with PEG hydrogel lines (PCL-Au-PEG).

### 2.1. Fabrication and Characterization of the Basic PCL Scaffold

**Figure 1:**
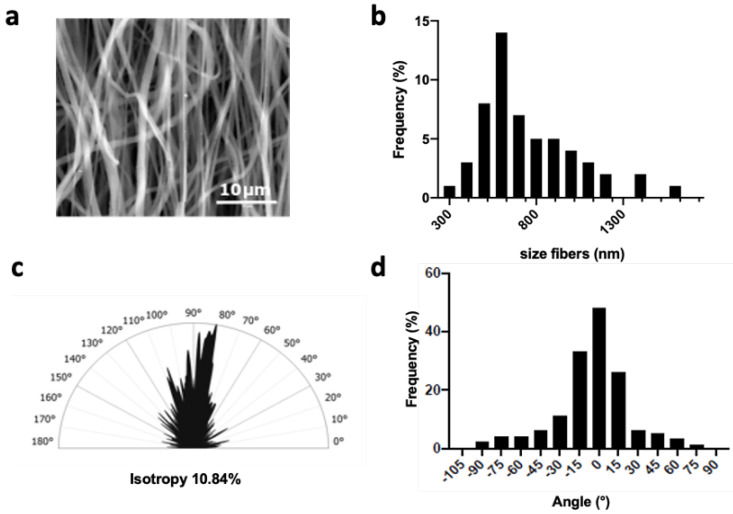
Characterization of electrospun fibers. (**a**) SEM images showing the electrospun fibers, (**b**) fiber size distribution (*n* = 100 fibers), (**c**) analysis of fiber orientations according to the principal directions using Fourier transform, and (**d**) half-polar smoothed curve of the orientation of the fibers from SEM image analysis (*n* = 3).

Our first objective was to produce a flat mat of partially aligned PCL fibers. A medium-range drum rotation speed of approximately 1000 rpm was thus applied during the electrospinning process. SEM images (Figure 1a) of the resulting PCL matrices showed smooth fibers with an average diameter of 776 ± 250 nm (Figure 1b), and a preferred orientation between −20° and 20° for 70% of them (Figure 1c,d). The wettability of the materials was assessed by the contact angle at each stage in the preparation for cell culture. First, the dry PCL electrospun sheet showed a contact angle (ϑ) of 130 ± 2.5°, corresponding to hydrophobic properties. After 45 min of ethanol treatment, the angle was about 115 ± 2.72°, the drop on the dry PCL scaffold retaining its shape with no change over time (data not shown). Finally, on PCL incubated for 30 min in culture medium and dried, the contact angle decrease to 72 ± 7° indicates hydrophilic interaction (Figure S1).

#### 2.1.1. Coating with Gold Nanoparticles (PCL-Au)

**Figure 2:**
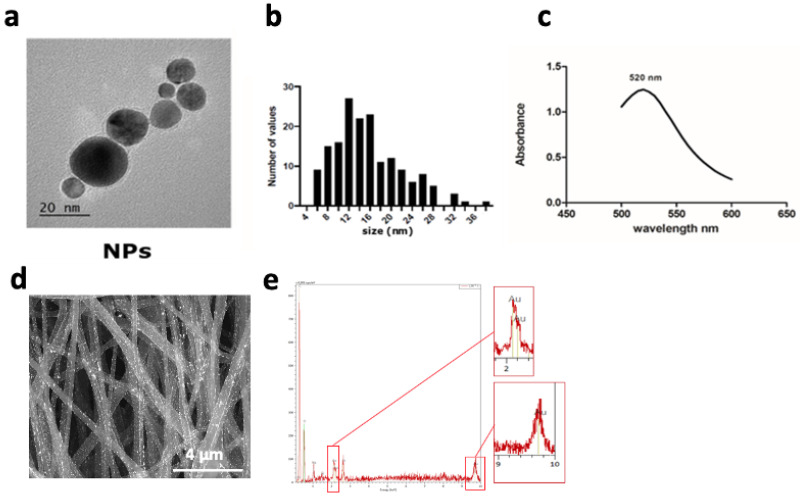
Characterization of the gold nanoparticles coated on the PCL scaffold. (**a**) TEM images of Au nanoparticles formed on the fibers, (presenting a s ize of 15 ± 6 nm ). (**b**) Particle size distribution from TEM image analysis (*n* = 30 particles) and (**c**) absorption spectrum of gold nanoparticle solutions using a spectrometer, with a peak of absorbance at a wavelength of 520 nm (*n* = 3). (**d**) SEM images of electrospun fibers coated with Au NPs, (**e**) EDS spectrum showing the 3 peaks for Au elements at 2.10, 2.20, and 9.7 keV.

To functionalize our synthetic fibers with AuNPs, we used an in situ chemical reduction method. The gold precursor solution was reduced with the help of sodium citrate, a reducing agent, and ascorbic acid, a stabilizer, in the presence of fibers to produce the Au-PCL nanocomposites [26,27]. During the reduction process of HAuCl_4_, we noticed that the white color of PCL nanofiber mats changed to purple, suggesting the formation of gold nanoparticles (Au NPs) (Figure S2). Scaffolds were then washed several times to remove any unbound nanoparticles and keep strong binding only. Nanoparticles of similar size and the detailed structures of single particles were observed in high TEM resolution images. Their mean diameter was estimated at 15.65 ± 6.41 nm (Figure 2b). In addition, EDS spectrometer analysis of the Au NP solution showed a peak at 520 nm (Figure 2c), confirming that the nanoparticles’ size was in the range of 15 to 20 nm. SEM and EDS were then used to assess the presence of the Au NPs on the electrospun fibers. SEM observation of the morphology showed that the Au NP coating did not obstruct the porous surface structure of the material. In the magnified SEM images of PCL-Au, white spots, relatively homogeneously dispersed along the nanofibers, were clearly observed (Figure 2d). The EDS profile of these spots presented strong gold atom signals around 2.10, 2.30 and 9.70 keV (Figure 2e). Polycaprolactone electrospun fibers covered with Au exhibited 3.5-fold lower resistance. However, the range of conductive values (2.10^−2^ S/cm) was not enough to be considered conductive (Figure S3).

**Figure 3:**
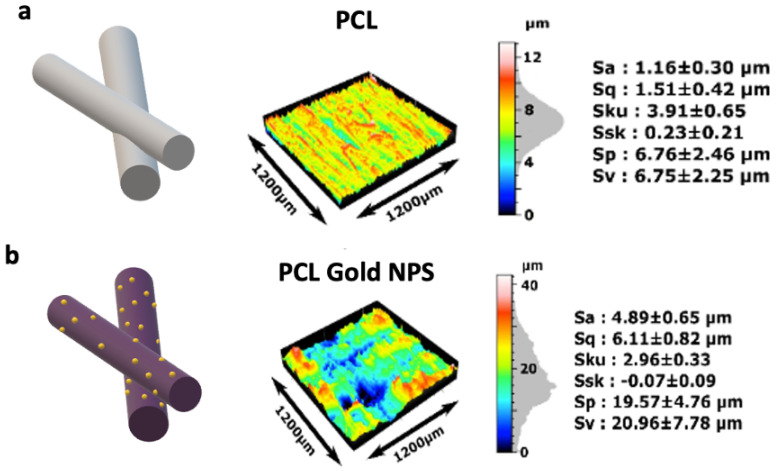
Topography of the PCL scaffolds coated or not with gold nanoparticles. Sensofar Confocal profiler measurement of the surface of (**a**) PCL and (**b**) PCL coated with gold nanoparticles. The features used to evaluate the surface roughness of the material were height parameters (Sv), valleys and peaks (Sp), including arithmetical mean height (Sa), and root mean square height (Sq). Skewness (Ssk) represents the degree of symmetry of the surface heights about the mean plane, while kurtosis (Sku) indicates the randomness of height and the sharpness of the structures on the surface (*n* = 3 for each condition).

The contact angle of the dry PCL-Au sheets was of 127 ± 6°, exhibiting similar properties as dry PCL sheets. After ethanol treatment, the measurement fell to 100 ± 9°. We observed that the drop was totally absorbed by the substrate before fifteen minutes after the deposit. Finally, the drops on dry PCL-Au incubated for 30 min in culture medium presented a contact angle of 38 ± 3.7° before being absorbed in less than a minute by the scaffold (Figure S1). Cell adhesion strength has been proven to vary logarithmically with increased surface roughness for polymeric and non-polymeric materials. Analysis with a non-contact 3D surface profilometer (Sensofar^®^) showed that the addition of nanoparticles to the fibers had an impact on the fiber’s roughness, with an increase in Sa (roughness average, arithmetical mean height) from 1.16 ± 0.30 µm to 4.89 ± 0.65 µm (Figure 3a,b). Although this parameter is often used to evaluate surface roughness, it is not enough to describe the complexity of a surface texture. The Sku (Kurtosis) characterizes the measurement of the sharpness of the roughness profile. Here, Sku was slightly higher for the scaffold without coating (Sku > 3). Ssk (skewness) centered around zero for both samples, suggesting that there is a similar proportion of peaks and valleys in the samples. Although surface topography with lower skewness (Ssk) and higher kurtosis (Sku) is known to exhibit better wettability, the variations in Sku and Ssk after the coating were not enough to significantly modify it.

#### 2.1.2. Micropatterning of Multiscale Scaffolds (PCL-PEG and PCL-Au-PEG)

**Figure 4:**
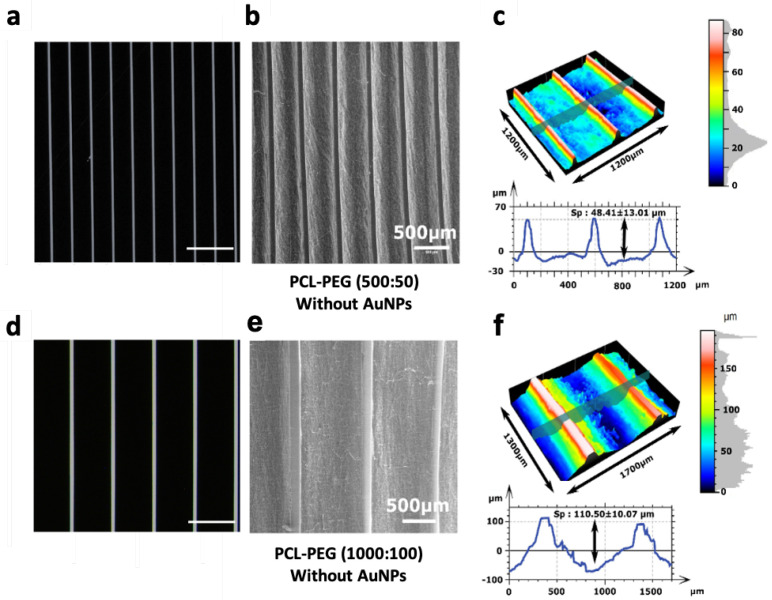
Characterization of the micropatterning of the scaffolds. (**a**,**d**) Images of the mask used for the photolithography of 50 µm and 100 µm PEG lines. (**b**,**e**) SEM images of line micropatterns with a spacing of 500 µm and 1000 µm respectively, on an electrospun mat. (**c**,**f**) Sensofar optical profiler measurements of the surface topography, height and thickness of the hydrogel line. Scale bar of panels (**a**,**d**) represents 1 mm and that of panels (**b**,**e**) 500 µm.

Two masks with linear patterns were selected to create PEG hydrogel lines (Figure 4a,d) on the previously produced scaffolds. They were spaced either 500 µm or 1000 µm apart, with a width of 50 or 100 µm, respectively (named 500:50 and 1000:100). Photolithography made correct PEG hydrogel line deposits possible and attached well to the electrospun fibers. SEM images (Figure 4b,e) demonstrated that the hydrogel microstructure, where deposited, fully covered the PCL structure, leaving no fibers exposed at the top surfaces of the micropatterns. No damage to the morphology of the fibers located in between the hydrogels was observed (Figure 4e). We noticed a difference by comparing the masked data with the corresponding polymeric replica obtained from the electrospun fibers. Profilometry measurements (Figure 4c,f) confirmed the SEM observations. The PEG lines in both patterns had a height of about 40 µm, but their widths showed an average of 108 µm and 124 µm instead of the expected 50 µm and 100 µm, respectively. This resulted in reduced mean spacing between two lines, of 377 µm and 921 µm for the 500:50 and 1000:100 patterns, respectively. However, this difference was significant enough to evaluate the effect of spacing on cell behavior. The Sensofar confocal analysis demonstrated that the sheet tends to bend between the PEG lines and this effect was more significant on the 1000:100 pattern, with a variation in height of 110.40 ± 9.9 µm, whereas it was only 44.46 ± 16.17 µm for the 500:50 pattern.

#### 2.1.3. Mechanical Properties of the Different Scaffolds

These investigations were conducted at two scale levels with different purposes.

**Figure 5:**
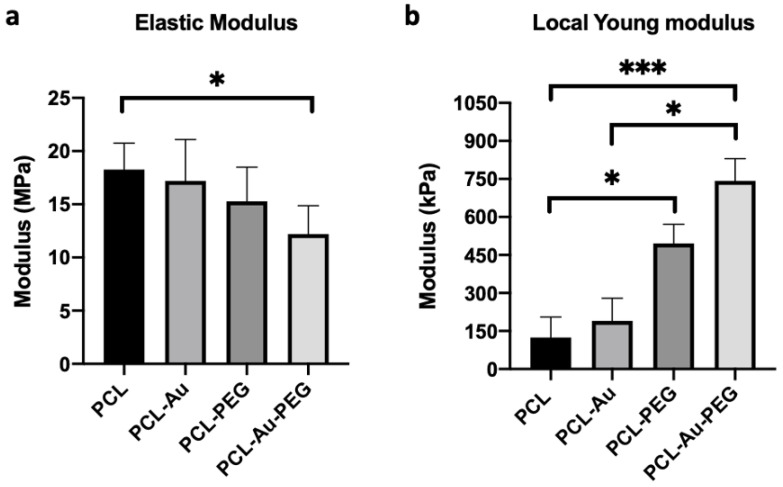
Mechanical characterization at the macro- and microscale levels. (**a**) Global elastic modulus obtained from the stress–strain curve in the tensile strength test on the four types of scaffold (*n* = 5) (**b**) Local Young modulus from nano-indentation. These data were compared with Dunn’s Multiple Comparisons Test: * *p* < 0.05 and *** *p* < 0.001. * indicates significant difference.

At the global scale, the issue is producing a scaffold that is easy to handle and capable of sustaining mechanical stretching cycles. Figure 5a shows the elastic moduli of the electrospun fibrous scaffolds before and after Au NP coating or hydrogel patterning. Based on the stress–strain curves, electrospun sheets coated or not with Au NPs display similar elastic moduli of 16 ± 2.5 MPa and 19.5 ± 3.0 MPa, respectively. After the addition of PEG hydrogel lines with a spacing of (500:50) on scaffolds, the mechanical properties slightly decreased to 14.8 ± 3.1 MPa for PCL-PEG and 12.2 ± 2.1 MPa for PCL-Au-PEG.

At the local scale, the properties of the electrospun fibers may affect cells’ response. Micro indentation was thus performed to access the local mechanical properties of the fibrous part of the different scaffolds (Figure 5b). The probe was followed by microscopy to ensure its localization on the fibers, and not on the PEG patterns. Surprisingly, the local moduli indicated a rather stiff material compared to the elastic moduli. Regarding the influence of PEG, the results were the opposite of those obtained for tensile strength. The local elastic modulus increased from 190.4 ± 96.9 kPa for PCL-Au to 764.3 ± 88.7 kPa when PEG micropatterning was added. The same trend was observed for the PCL scaffold: the local modulus increased from 124.6 ± 32.4 kPa to 495.7 ± 71.8 kPa with the PEG.

#### 2.1.4. Influence of Material Composition on Adhesion, Proliferation, and Cell Viability

**Figure 6:**
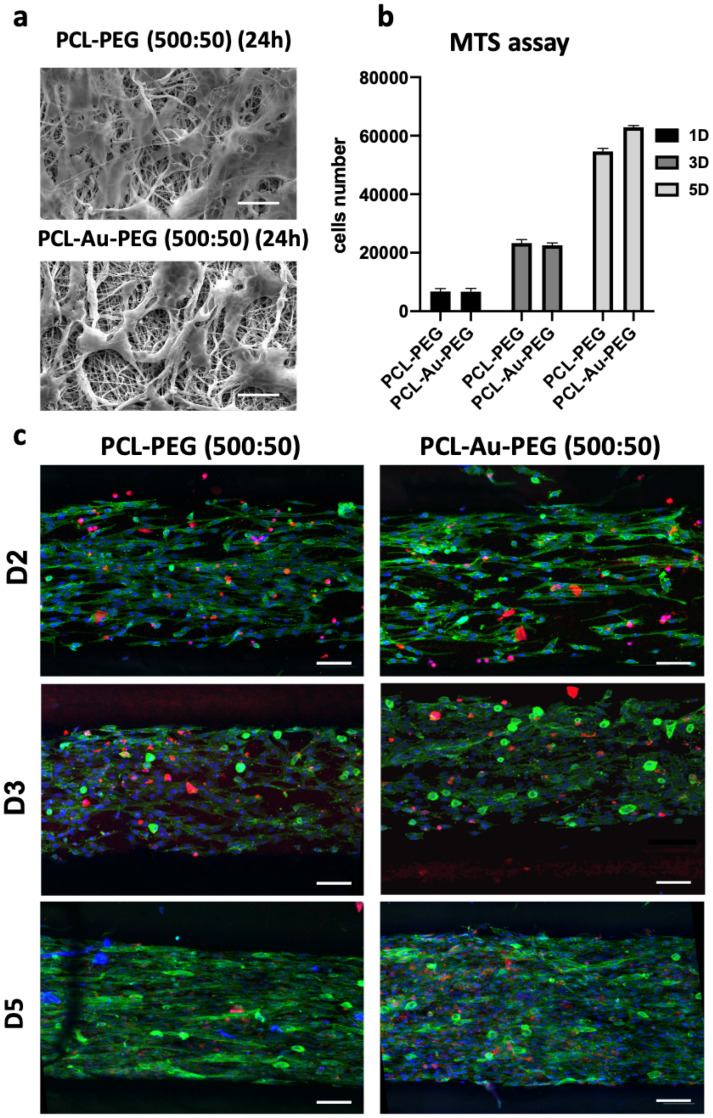
Cell adhesion and proliferation on the scaffolds. (**a**) MEB images of C2C12 myoblasts adherent to the scaffolds with micropatterned (500:50) coated or not with gold nanoparticles, 24 h after cell seeding. Scale bar 20 µm. (**b**) MTS assay illustrating cell proliferation was followed on PCL-PEG (500:50) and PCL-Au-PEG (500:50) at D1, D3, and D5 (*n* = 6); (**c**) the viability was observed on those scaffolds by confocal images with dead cells emitting in red fluorescence (Far Red), nuclei in blue fluorescence (Hoechst-33258) and F-actin in green fluorescence at 2, 3, and 5 days. Scale bar represents 100 µm. (The data obtained for MTS were compared with the Mann–Whitney nonparametric statistical test: there was no statistical difference between the conditions PCL-PEG (500:50) and PCL-Au-PEG (500:50).

Cell adhesion was investigated on (500:50) PCL-PEG and PCL-Au-PEG. SEM images (Figure 6a) show C2C12 cells attached and distributed on the scaffolds at 24 h, which confirmed that both scaffolds could support the attachment and proliferation of myoblasts. Cell proliferation assay revealed that the cells displayed the same growth characteristics on the different materials, with threefold growth (Figure 6b). A difference appeared on D5, with greater proliferation of cells on the material with gold nanoparticles. A Far Red assay was performed to investigate the viability of the C2C12 cells on the composite materials (Figure 6c). After 5 days it was evaluated that 13.2 ± 3% and 20.8 ± 5.5% of the C2C12 cells were dead (red staining) on PCL-PEG and PCL-Au-PEG scaffolds, respectively. These tests indicate that neither substrate induced any cytotoxicity. On these confocal observations, cell proliferation and changes in shape could also be followed from day 2 to day 5.

#### 2.1.5. Cell Differentiation

**Figure 7:**
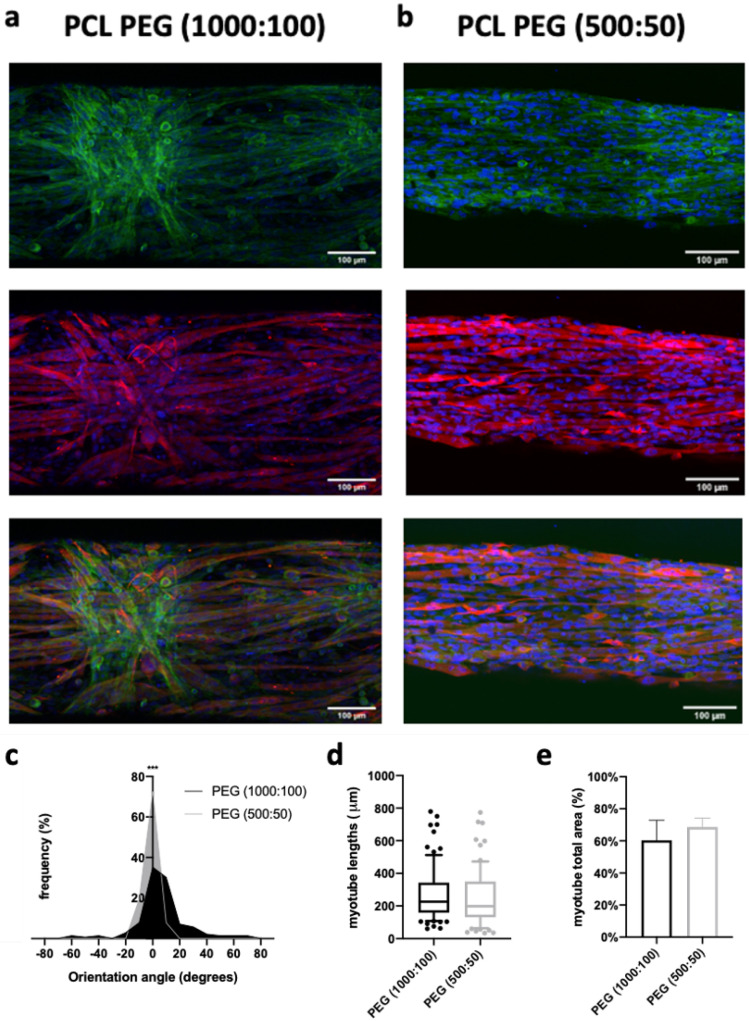
Myotube morphology on the scaffolds. Confocal images of C2C12 cells after 7 days of culture on scaffolds coated or not with gold nanoparticles with a space of (**a**) 1000 µm or (**b**) 500 µm between the hydrogel lines. Nuclei in blue fluorescence (Hoechst 33258), F-actin in green fluorescence and myotubes by anti-MHC antibodies in red fluorescence. Scale bar: 100 µm. Quantification of the (**c**) orientation (significance analyzed by Mann–Whitney nonparametric two-tailed test), (**d**) length and (**e**) total area occupied by the myotubes as a function of the space patterning (*n* = 50 myotubes). There was no statistical difference between the conditions of the length and area of the myotubes. *p* < 0.001. *** indicates significant difference.

Finally, we evaluated the effects of spacing micropatterns on PCL scaffold (500:50 vs. 1000:100) on the differentiation of C2C12 myoblasts into myotubes, as well as on their spatial orientation. On day 7, cells were immunostained with myosin heavy chain (MHC), an indicator of myotube formation. Confocal microscopy images confirmed that under partial serum deprivation (2% of horse serum) for 5 days, myoblasts fused into myotubes on the scaffolds (Figure 7a,b). Moreover, confocal images of fluorescent stained F-actin on cells demonstrated that the actin assembly appeared perfectly oriented along the fibers and pattern structure for the PEG samples (500:50). In contrast, in some parts of the PEG sample (1000:100), on the apparent strata of cells, the actin could appear disordered. Yet, it was interesting to observe that those cells were not in direct contact with the PCL fibers, but lay on other cells and tended to assemble and arrange into spiral-like patterns (Figure 7a). In comparison, the cells in direct contact with PCL nanofibers formed elongated myotubes assembled into parallel patterns. Muscle-like cells were found to be arranged mostly in parallel with the patterned hydrogel. For the PCL-PEG (500:50), i.e., the narrow configuration, all angles formed by the myotubes with the direction of the PEG lines were found in the range −15°/ + 15°, with a mean value of −0.9° ± 5. When the space between two PEG lines increased (PCL-PEG 1000:100), only 72% of the angles were in this range, with a mean value of 7.35° ± 21.95° (Figure 7c). Analysis of the confocal images in (Figure 7d) showed that the length of the myotubes was not significantly higher with the wider spacing patterns. Very interestingly, some myotubes presented lengths in the range of 800 µm. Finally, myotubes that formed on PCL-PEG (500:50) covered 8% more surface area than on the PCL-PEG (1000:100) (Figure 7e). In both cases, surface coverage was very high. One limitation of electrospun scaffold regarding direct cell observation comes from its opacity: it was not possible to evaluate if C2C12 were able to spontaneously contract, as is commonly observed in plastic culture dishes.

**Figure 8:**
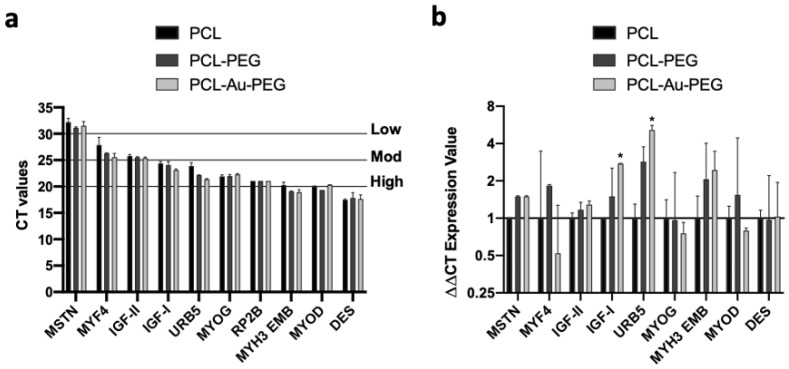
RT-PCR analysis on bio-constructs. A one-week-old construct made of C2C12 cells on electrospun PCL with PEG hydrogel (500:50) and electrospun PCL coated with gold nanoparticles and PEG hydrogel (500:50) were cultured. The constructs were analyzed for 9 genes that regulate myoblast differentiation and myotube maturation: myostatin, myf4, IGF-I/II, URB5, myogenin, myosin heavy chain 3 embryonic (MYH3 EMB), myoD, and desmin by means of RT-PCR analysis. (**a**) The basal Ct values were between 20 and 25 for 8 out of 10 genes, indicating a high to moderate gene expression profile. (**b**) The ΔΔCt expression values were compared with the Mann–Whitney nonparametric statistical test: *p* < 0.05 * indicates significant difference.

Finally, to assess the terminal differentiation of the C2C12 myoblasts on the PCL constructs we analyzed nine genes that regulate myoblast differentiation and myotube maturation. These corresponded, from the least to the most matured status, to myostatin (MSTN), myf4, IGF-I/II, myoD, URB5, myogenin (MYOG), myosin heavy chain 3 embryonic (MYH3 EMB), and desmin. The basal Ct values suggest that eight out of 10 genes (including the reference gene: RP2b) were between 20 and 25, indicating a high to moderate gene expression profile (Figure S4). Myostatin, a negative regulator of muscle mass, displayed the lowest expression levels for all treatments, and desmin expression was the highest (Figure 8a). Interestingly, the highest tertile of expression comprised myogenic regulatory factors and embryonic myosin, which also increased during regeneration. Next, the ΔΔCt expression values were quantified, following normalization with the basal Ct values of the housekeeper gene (RP2b) and the experimental control (PCL statical) at 7 days (Figure 8b). There was no difference in myostatin expression between PCL-PEG and PCL-Au-PEG constructs vs. control. Myf4 expression increased non-significantly by 2.9 and 3.6 in PCL-PEG vs control and PCL-Au-PEG, respectively. There was no difference in IGF-II expression across experimental conditions. However, there was a significant, 2.5-fold increase and a small, but non-significant 1.9-fold increase in IGF-I expression in PCL-Au-PEG vs. control and PCL-PEG respectively. Strikingly, there was a non-significant 2.8-fold and a significant 5.1-fold increase in URB5 expression vs. control. Further, there was 1.8 times greater URB5 expression in PCL-Au-PEG vs. PCL-PEG, although significance was not achieved. Despite small 1.5 and 1.8-fold increases in MYH3 EMB expression in PCL-PEG and PCL-Au-PEG vs. control, significance was also not achieved. Lastly, there was no impact of treatment on the myogenic regulatory factors Myogenin, MyoD, or desmin. However, these genes were among those with highest expression basally (basal Ct values).

## 3. Discussion

To date, many attempts have been made in tissue engineering to reconstruct skeletal muscle tissue in vitro. Current consensus states that controlling myoblast orientation is essential for achieving successful regulation and differentiation of skeletal muscle cells in vitro [5,25,28]. Most studies have thus focused on designing appropriate scaffolds that mimic the structure of native tissue to support the formation of highly oriented and functional myofibers. Bioinspiration could be found at the nanoscale level (mimicking the size and orientation of the matrix fibers) or at the microscale level (guiding the direction of cell proliferation). However, other key aspects of bioengineering cannot be neglected, such as the handling of the biohybrid construct, and the ability to perform dynamic stretching to promote cell differentiation. This required the production of scaffolds with adequate mechanical resistance. Here, we investigated a multicale approach to meet all of these requirements. The scaffold contains three elements: the electrospun nanofiber sheet where the cells are seeded, gold nanoparticles for conductivity, and hydrogel micropatterning to confine the cells in a defined area on the nanofiber sheet and thus lead to optimized muscle fiber formation. The PCL electrospinning process was tuned to produce matrices with fiber diameters of around 700 nm, to be similar to individual myofibrils (1 µm) [29,30]. Using a drum collector rotating with a tangential speed of 3.9 ms${}^{-1}$ resulted in 70% parallel-aligned fibers, which made it possible to guide most of the cells towards one direction.

PCL is also known to be highly hydrophobic and can present poor cell attachment in vitro [31]. Therefore, the fibers were coated with gold nanoparticles to increase the wettability of the scaffold, to implement electrical conductivity properties and the possibility to functionalize with biological peptides or proteins such as collagen to improve the adhesion and proliferation of the cells on the polymeric electrospun mats [32]. PCL-Au sheets showed higher hydrophilic properties than the PCL alone after all treatment applied (Supplementary Data) [33]. However, the quantity of gold nanoparticles on the surface of the electrospun fibers did not make it possible to bring conductive properties to the scaffold. These nanoparticles were found in too small quantities and thus scattered on the whole fibrous sheet. Au vaporization could be considered as another method for maximizing deposits of gold particles on the fibers [34]. An alternative approach is co-electrospinning PCL with electronic conductor polymers such as polyaniline [35].

Regarding the mechanical properties of the substrates, most electrospun matrices described in the literature show elastic moduli in the range of tens or hundreds of MPa [36]. In this study, the elastic modulus of the electrospun scaffold was reduced to a range of 10 to 20 MPa, values that remain very high compared to the elasticity of native tissues (10–50 kPa) [37,38,39]. Interestingly, the presence of PEG lines modified the mechanical properties of the scaffold, mostly at the microscale level, where a 4-fold increase in the surface’s elastic modulus was measured. It was hypothesized that some PEG residues were still present and cross-linked within the electrospun mat when exposed to surrounding UV light, thus trapping the fibers deep inside and modifying the properties of the material. PCL biodegradability could ultimately contribute to decreasing the mechanical properties of the scaffold to a physiological range and promote neotissue formation [40,41]. However, this raises the question of the different rate of biodegradability of the PCL and PEG [42,43,44]. In addition, the degradation of biomaterial polymers substrates, and thus the release of low doses of nanoparticles in vivo, could trigger a classical foreign body reaction pattern and induce the nonspecific immune response [45,46,47,48,49].

As already stated, in the present study nanoscale and microscale levels were investigated simultaneously to facilitate the alignment of the cytoskeleton and the formation of myotubes. As myofibers can reach a few hundred microns in diameter, a linear pattern with PEG hydrogel (40 µm height, 50 or 100 µm wide) spaced 500 to 1000 µm apart was developed. The multiscale scaffolds designed were successful for myoblast adhesion, proliferation, and most importantly fusion and differentiation into myotubes. The patterns with 500 µm to 1000 µm spacing positively affected cell organization and alignment, when coupled with a nanofibrous structure. Furthermore, the use of pattern spacing up to 500 µm promotes the development of myofibrils in the physiological range which can subsequently bundle together to form muscle fibers. Very interestingly, the surface coverage was also much larger than in most previous studies with micro-structured hydrogel implemented patterns that created interspaces from 5 to a few hundred micron [25,50,51]. A decrease in cell alignment for interspaces of more than 200 µm has been reported in the literature [26,52].

Here, by coupling the nanopatterning from the fibers and the hydrogel micropatterning, the myoblasts jointly “sensed” the topographical barrier imposed by the micropattern and the nanofiber direction and developed highly organized stress fibers along the pattern axis. In fact, studies have already demonstrated the complementarity and advantages of combining these different scales. Previous work has established a method for patterning multiple desired topographic surfaces on electrospun nanofibers via solvent-loaded agarose hydrogel stamps [53].

In addition, through a hierarchical patterned topography of microgroove and nanopore structures hNSCs was successfully directing into neuronal differentiation [54]. In the same way, skeletal muscle cell behaviors on nano- and micro-alignment scaffolds combined with different angular combinations were monitored [55].

To go further than these morphological observations, we monitored the behavior of cells through the expression of specific genomics markers. The expression of the myosin heavy chain, a mature muscle marker, demonstrated that the multiscale scaffold allowed the C2C12 myoblasts to differentiate into myotubes. Maximizing the formation of dense and cohesive myotubes promoted their fusion to form larger myofibers. The cell behavior observed in these experiments was corroborated by the increase in expression of various genes selected because they are known to play a part in myogenesis. To support the morphological and structural data, 9 genes that regulate myoblast differentiation, myotube maturation and regeneration were analyzed. Only myostatin, which is a negative regulator of myoblast differentiation following downregulation of MyoD expression [56] displayed very low expression, whereas the remaining eight genes that positively regulate myogenesis and muscle growth were all expressed at moderate to high levels, based on raw Ct values. After normalization, both the PCL-PEG and PCL-Au-PEG constructs displayed similar myostatin expression vs. control PCL. The myogenic regulatory factors myogenin, myf4 and myoD are essential for early and late myoblast differentiation [57]. Interestingly, it appears that the PCL-PEG construct increased myf4 expression compared to control PCL and PCL-Au-PEG, suggesting increased differentiation at the final stages for myogenesis. MyoD is critical for cell cycle exit and commitment to differentiation [58] and myogenin throughout differentiation. There was no effect of either PCL-PEG or PCL-Au-PEG construct at 7 days on myogenin or myoD expression, suggesting that the process of myotube formation had occurred in all constructs, supporting the morphological data. The growth factors IGF-I/II also regulate late differentiation [59,60,61]. Although highly expressed, there was no effect of PCL-PEG or PCL-Au-PEG construct compared to control on IGF-II expression. Whereas IGF-I gene expression was increased with the nanoparticles construct compared to control and also PEG. High expression of IGF-I also improves muscle hypertrophy by supressing the atrophy-related ubiquitin ligases, atrogin-1 and MuRF1 [62]. This suggests that following the formation of myotubes in all constructs, the subsequent elevation of IGF-I expression in the NPS construct could drive hypertrophy compared to control PCL and PCL-PEG at 7 days. MYH3 EMB is also essential in development and regeneration. Overexpression results in increased myofiber size knockdown reduces fusion capability [63].

Both PCL-PEG and to a greater extent PCL-Au-PEG constructs increased MYH3 embryonic expression, suggesting increased myotube growth or potential for regeneration. Finally, URB5, an E3 ligase gene, helps control myogenin stability which is necessary for terminal differentiation in skeletal muscle progenitors. However, it also has a primary role in regulating muscle hypertrophy, recovery from atrophy and remodeling [64]. There were significant increases in URB5 expression with both PEG and to a greater extent with nanoparticle constructs compared to control at 7 days. Together with IGF-I data, this suggests that the C2C12 myotubes demonstrated greater hypertrophyin the PCL-Au-PEG construct, which is rather positive in a tissue engineering process. Overall, these gene expression data demonstrate that the constructs allow effective and efficientmyogenesis and also increasedmyotube size, particularly with the PCL-Au-PEG construct at 7 days.

In our bioinspired approach, the next step towards the improvement of cell differentiation on the scaffold will consist in implementing physical stimuli (either mechanical or electrical). It would be then interesting to evaluate if such stimuli will be synergetic or antagonist, according to the different topography. We believe that such constructs will definitely be useful for in vitro investigations, regarding the effects of drugs for instance, or for developmental studies. For in vivo application, 3D constructs should be preferred, using human cells, and would request to be treated as advanced therapy medicinal products.

## 4. Materials and Methods

### 4.1. Scaffold Preparation and Characterization

**Figure 9:**
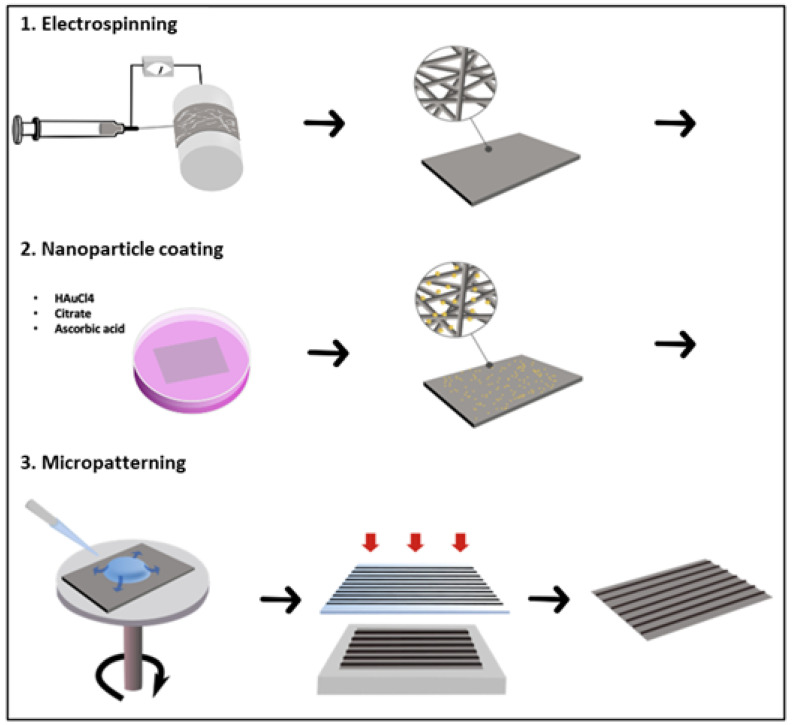
Schematic illustration showing the three steps in the manufacturing process: (1) electrospinning of PCL on drums at high velocity; (2) the nanoparticle coating process, including the successive addition of gold [HAuCl_4_], citrate, and ascorbic acid solutions to the scaffold immersed in milliQ water; and (3) soft photolithography of polyethylene glycol (PEG) hydrogel, with 50 µm or 100 µm width line patterning and spaced 500 µm or 1000 µm.

Three steps were necessary to obtain the multiscale scaffold (Figure 9).

**Step 1: Preparation of electrospun PCL** A solution of 10 wt% poly(ε-caprolactone) (PCL, MW = 80.000 Da, Sigma-Aldrich, St. Louis, MO, USA) in dichloromethane (DCM, Sigma-Aldrich)/*N*,*N*-dimethylformamide (DMF, Reagent Plus ≥99%, Sigma-Aldrich) (80:20 *v*/*v*) was prepared under stirring for 24 h before electrospinning. Polymer solutions were loaded into a 5 mL syringe equipped with a (18 G) stainless steel gauge needle. Grounded aluminum foil was used as the collector electrode. The distance between needle and aluminum collector was 15 cm and the collector had a diameter of 75 mm. Solution was fed in constantly using a syringe pump at 1.02 mL/h. The voltage applied was optimized to obtain good spinnability, with a typical value of 15 kV.
**Step 2: Preparation of Au NP-doped PCL nanofibrous scaffolds** Gold nanoparticles were prepared in surfactant solutions by reduction of HAuCl_4_. The electrospun scaffolds were immersed in 2 mL of distilled water, successively in a solution of chloroauric acid HAuCl_4_ (20 mM Sigma-Aldrich). Citrate (70 mM) and ascorbic acid (17.6 mg/mL) were added as reducing agent and stabilizer. The reaction was allowed under stirring for 1 h, during which the color of the scaffolds changed to purple (Supplementary Data S1). Subsequently, the Au NP-doped nanofibrous scaffolds formed were rinsed three times with deionized water and vacuum dried at room temperature for 24 h.
**Step 3: Preparation of (PEG) hydrogel micropatterns on PCL nanofibrous scaffolds** The resulting electrospun fibers (with and without Au NPs) were micropatterned with PEG hydrogel using photolithography. PEG-diacrylate (MW 575) was purchased from Sigma-Aldrich. For the UV photo-crosslinking process, the liquid PEG was mixed with 1% *w*/*v* of photo-initiator (2-hydroxy-2-methylpropiophenone, Darocur 1173, Sigma Aldrich). Then the mixture was dropped on electrospun scaffolds by spin coating (SPINCOATER model P6700) and exposed to a UV light source for 20 s (Kloé UV-KUB 2, 365 nm, 40 mW/cm${}^{2}$) through a photomask. The patterned scaffold was washed carefully in the dark with distilled water to remove the PEG precursor solution.

The topography of the electrospun scaffolds was observed using environmental scanning electron microscopy (Philips XL30 ESEM-FEG). Fiber diameter was measured after setting up the scale bar. Average fiber diameters (*n* = 100 fibers) were analyzed with ImageJ software (NIH, Bethesda, Maryland). The isotropy value of the scaffold (*n* = 6) was analyzed using Mountain™ version 8.0 software (with smoothing and maintaining the default frequency thresholds at 5% and 80% Str ISO 25178) and the main orientations of the fibers were analyzed using the Fourier Transformation method (*n* = 6). Gold deposits over the electrospun scaffold were investigated using Energy Dispersive X-ray Spectroscopy (EDS) analysis with the detector present in the microscope. The measurement is based on the energy and intensity distribution of X-ray signals produced by the electron beam striking the surface of the target scaffold. Samples for transmission electron microscope (TEM) analysis were prepared by dropping a dilute suspension of gold nanoparticles on to copper grids covered by a carbon film. Grids were observed with a Philips Tecnai 12 transmission electron microscope.

### 4.2. Scaffold Conductivity

Electrical conductivity was measured by a source meter (model 2602-A, Keithley Instruments, Inc., Cleveland, OH, USA) using a two-point probe method at room temperature. The electrical conductivity (σ) of AuNPs scaffolds and AuNPs-PEG scaffolds were obtained using the resistance (R) obtained between the 2 plates the surface area (S) and the distance (l). the conductivity σ is found using the following equations: σ = 1/R.

### 4.3. Contact Angle Measurement

A standard static sessile drop method (VCA Optima XE, VCA Optima XE, AST Products Inc., Billerica, MA, USA) was used to characterize the wettability of the electrospun scaffolds. A 10 µL water droplet was dropped on to the surface of the scaffold (*n* = 3) and a side-view photo was taken to measure the contact angles. The measurement was repeated twice (Supplementary Data).

### 4.4. Mechanical Properties

The mechanical properties of the scaffold were evaluated with different devices depending on the scale of the study.

The whole scaffold’s elastic modulus was quantified using uniaxial tensile testing. One sample of each scaffold (*n* = 3) was cut into a strip measuring 1.0 × 3.0 cm, with a thickness of 100 ± 10 µm. The thickness of the scaffolds was evaluated using a precision dial thickness gauge (Mitutoyo Corporation, Kawasaki-shi, Japan). The samples were secured with the metallic grips of the tensile tester (Bose Electroforce 3200, TA, Eden Prairie, MN, USA) and stretched at a rate of 0.05 mm·s^−1^ using a cell load of 22 N. The elastic modulus was calculated by analyzing the recorded stress–strain curve in the elastic zone, where the relationship is linear, i.e., generally between 5 and 10% strain.

The local Young modulus (E) of each scaffold was measured with a Chiaro nanoindenter system (Optics 11, Amsterdam, The Netherlands) mounted on an optical microscope. Two probes were selected based on estimation of the materials’ stiffness and the manufacturer’s probe selection chart. The probes selected were 5.23 N/m with a tip radius of 9 µm for mats without PEG, and with a spring constant of 47.69 N/m and a radius of 25 µm for mats with PEG hydrogel. Before testing, the optical sensitivity and geometrical factors were calibrated by indenting a hard surface (e.g., a glass slide). A fibrous mat was deposited on a glass slide and the probe was placed in contact with the scaffold where an indentation of 15 µm was made. All experiments were performed at room temperature. For each condition, about 9 curves were acquired. Data were analyzed with DataViewer 2.2 Software (Optics 11, Amsterdam, The Netherlands) using the Hertzian contact theory to calculate the local E.

### 4.5. Profilometry

The surface structure of the different samples was measured using an optical profilometer laser (Sensofar) on 3 samples (PCL, Au NPs PCL, and micropatterned scaffold) using a 1746 × 1313 µm zone. For each specimen, 3 measures were performed to extract roughness features: height parameters (Sv), and valleys and peaks (Sp), including arithmetical mean height (Sa), root mean square height (Sq), and maximum height (Sz). Skewness (Ssk) is the degree of symmetry in the surface heights about the mean plane, while kurtosis (Sku) indicates the randomness of height and the sharpness of the structures on the surface. Specimens were also examined with SEM (accelerating voltage of 20 kV) to analyze fiber texture after the addition of hydrogel.

### 4.6. Cell Seeding on Scaffolds

Murine C2C12 skeletal muscle myoblast cells (ATCC CRL-1772) were cultured on T-75 flasks at 50% of confluence with growth medium, Dulbecco’s Modified Eagle’s Medium high-glucose (HDMEM; Hyclone, Logan, UT, USA) supplemented with 10% fetal bovine serum (FBS, Gibco Invitrogen, Logan, UT, USA) and 1% of penicillin–streptomycin (Gibco Invitrogen). To evaluate the response of the cells to materials, the scaffolds were cut into rectangles measuring 30 mm × 10 mm, disinfected with ethanol 70% (Sigma-Aldrich, Waltham, MA, USA) for 45 min, washed three times with PBS (phosphate buffered saline, Gibco Invitrogen, Logan, UT, USA) pH 7.4 and incubated in growth media for 30 min before starting the cell culture in 6-well plates. Each scaffold was plated with a density of 5 × 10^3^ cells for viability and proliferation tests and 5 × 10^5^ cells for the confocal analysis. With the highest density, after 48 h, culture was ~80% of confluence. Then, the growth medium was changed to differentiating media constituted of HDMEM supplemented with 2% horse serum (HS, Gibco Invitrogen, Logan, UT, USA) and 1% of penicillin–streptomycin (Gibco Invitrogen, Logan, UT, USA) and culture was prolonged for five more days.

### 4.7. Evaluation of Cell Adhesion, Viability, and Proliferation

#### 4.7.1. Adhesion

On day 1, the cell-seeded scaffolds were removed from the culture medium, gently washed with PBS and soaked in a buffered 4% paraformaldehyde (PFA) solution (VWR) prior to observation using scanning electron microscopy (SEM) (XL 30-ESEM FEG, Philips, The Netherlands) to evaluate the cells’ attachment and growth.

#### 4.7.2. Viability

On days 2, 3 and 5, cell viability was estimated with the Far-Red fixable dead cell staining Kit (ViaQuant™, GeneCopoeia, Rockville, MD, USA). The samples were observed using fluorescence microscopy at a wavelength of 650 nm (Leica Microsystem, Wetzlar, Germany), making it possible to determine cell viability and distribution.

#### 4.7.3. Proliferation

CellTiter 96^®^AQueous One Solution Cell Proliferation Assay (Promega, Madison, WI, USA) was used to evaluate the cell proliferation at different time points (days 1, 3, and 5). One-hundred microliter MTS solution in complete culture media were added to each well (*n* = 6). After 4 h of incubation at 37 °C, the absorbance of the solution was measured using a Spark multimode microplate reader (TECAN, Männedorf, Switzerland) at a wavelength of 570 nm and was then recorded with a 96-well plate reader. Finally, cell behavior after 7 days of culture on the scaffold was assessed through immunofluorescence. After washing with PBS, the samples were fixed in a 4% PFA solution for 10 min at room temperature. Samples were permeabilized with a 0.2% TritonX-100 solution for 10 min and blocked with a 2% bovine serum albumin solution (BSA, Sigma) for 30 min. Myosin, actin, and nuclei staining of cells was performed, using myosin heavy chain antibodies (1/200, Neo Biotech, Seoul, Republic of Korea) for 2 h followed by secondary staining using Alexa 594 overnight (1/200, Thermofisher, Waltham, MA, USA), prior to Alexa Fluor 488 Phalloidin (1/200, Thermofisher) staining for 2 h and Hoechst 33258 (1/1000, Sigma) for 15 min. The samples were finally washed with PBS before visualization using Z-stacking and mapping “Tile scan” specifications of confocal microscopy (Zeiss LSM 710). The confocal images were about 1 mm in size.

### 4.8. Myotube Measurement

The influence of the biomaterials on myoblast differentiation, myotube length, orientation and total area occupied was measured using ImageJ (NIH, Bethesda, MD, USA) and Cell ProfilerTM (Broad Institute, Cambridge, MA, USA) software [65]. Myotube length was defined as the line distance from one extremity of the myotube to the other. The total area occupied by differentiated cells was counted in 3–5 pictures, selecting an area of 2 mm × 2 mm for each sample. The percentage of cell alignment was defined based on the measurement of myotubes (*n* = 100) aligning with ± 20° from the pattern.

### 4.9. RT-qPCR

Gene expression was studied using RT-qPCR (reverse transcription quantitative polymerase chain reaction) after 7 days of culture on the scaffolds. Briefly, samples were lysed with 350 µL of Trizol and centrifuged to extract the RNA (ribonucleic acid) according to the manufacturer’s protocol (Qiagen, Hilden, Germany). The RNA was retrotranscribed into DNA (deoxyribonucleic acid) using a High Capacity cDNA Reverse Transcription kit (Applied Biosystems, Waltham, MA, USA) according to the manufacturer’s protocol. RT-qPCR was performed using the SYBR Green PCR Master Mix (Applied Biosystems). Relative mRNA levels were calculated using the ΔΔCt method. The ΔCts were obtained from Ct normalized with the Rp2β, gene levels in each sample and reactions were checked before the experiments (efficiency > 80%, R2 > 0.99). The results were normalized with the data from the control for this experience, the basic PCL electrospun construct, to highlight the intrinsic effect of the scaffolds’ modifications on gene expression. The primers used are listed in the Supplementary Information (Table S1).

### 4.10. Statistical Analysis

Statistical analysis and graph drawing were carried out using GraphPad Instat 3.10 and Prism v 6.0 (GraphPad Software, San Diego, CA, USA). All data are represented as mean ± standard deviation from at least three independent cultures (*n* ≥ 3). Group comparisons were performed using the Mann–Whitney nonparametric two-tailed test and the Kruskal–Wallis nonparametric test with Dunn’s multiple comparisons post-test. Significance is indicated on the graph by * *p* < 0.05; ** *p* < 0.01; and *** *p* < 0.001.

## 5. Conclusions

In this study, we presented a simple and direct approach to control cellular alignment and elongation in a tissue engineered construct. We propose a hybrid method for manufacturing a multiscale scaffold. By using a simple photolithography process on the electrospun scaffold, we obtained an effective micropatterned polymeric surface. We performed an analysis of C2C12 cell behavior on two types of substrate, coated or not with gold nanoparticles and two ranges of patterning. Correct cell attachment and tissue formation were obtained in each substrate. Moreover, cell alignment was induced simultaneously by the nanofibers and linear micropatterning. The best results for differentiation parameters were observed on the scaffold coated with a micropatterning spacing of 500 µm. Immunofluorescence analysis indicated the formation of myotubes, corroborated by gene expression. Further studies could enhance by investigating the effect of different external stimuli, such as electrical or mechanical stimulation.

## Data Availability

Not applicable.

## Supplementary Materials

The following are available at https://www.mdpi.com/article/10.3390/ijms23010260/s1.

## Abbreviations

The following abbreviations are used in this manuscript:

AuNPs	Gold nanoparticles
E	Local Young modulus
hNSCs	Primary Human Neural Stem Cells
MYOG	Myogenin
MHC	Myosin heavy chain
MTS	3-(4,5-dimethylthiazol-2-yl)-5-(3-carboxymethoxyphenyl)-2-(4-sulfophenyl)-2H-tetrazolium)
MYH3 EMB	Myosin heavy chain-embryonic
MSTN	Myostatin
PCL	Poly(ε-caprolactone)
PDMS	Polydimethyl-siloxane
PEG	Polyethylene Glycol
Sa	Arithmetical mean height
Sku	Kurtosis
Sp	Maximum peak height
Sq	Root mean square height
Ssk	Skewness
Sv	Maximum pit height

## References

[1] Juhas M., Bursac N. (2013). Engineering skeletal muscle repair. Curr. Opin. Biotechnol..

[2] Shadrin I.Y., Khodabukus A., Bursac N. (2016). Striated muscle function, regeneration, and repair. Cell. Mol. Life Sci. CMLS.

[3] Frontera W.R., Ochala J. (2014). Skeletal Muscle: A Brief Review of Structure and Function. Calcif. Tissue Int..

[4] Sosa H., Popp D., Ouyang G., Huxley H. (1994). Ultrastructure of skeletal muscle fibers studied by a plunge quick freezing method: Myofilament lengths. Biophys. J..

[5] Wakelam M.J. (1985). The fusion of myoblasts. Biochem. J..

[6] Cheng Y.W., Shiwarski D.J., Ball R.L., Whitehead K.A., Feinberg A.W. (2020). Engineering Aligned Skeletal Muscle Tissue Using Decellularized Plant-Derived Scaffolds. ACS Biomater. Sci. Eng..

[7] García-Lizarribar A., Fernández-Garibay X., Velasco-Mallorquí F., Castaño A.G., Samitier J., Ramon-Azcon J. (2018). Composite Biomaterials as Long-Lasting Scaffolds for 3D Bioprinting of Highly Aligned Muscle Tissue. Macromol. Biosci..

[8] Charest J.L., García A.J., King W.P. (2007). Myoblast alignment and differentiation on cell culture substrates with microscale topography and model chemistries. Biomaterials.

[9] Jiao A., Moerk C.T., Penland N., Perla M., Kim J., Smith A.S.T., Murry C.E., Kim D.H. (2018). Regulation of skeletal myotube formation and alignment by nanotopographically controlled cell-secreted extracellular matrix: Regluation of myotube formation by matrix nanotopography. J. Biomed. Mater. Res. Part A.

[10] Xu B., Magli A., Anugrah Y., Koester S.J., Perlingeiro R.C.R., Shen W. (2018). Nanotopography-responsive myotube alignment and orientation as a sensitive phenotypic biomarker for Duchenne Muscular Dystrophy. Biomaterials.

[11] Choi J.S., Lee S.J., Christ G.J., Atala A., Yoo J.J. (2008). The influence of electrospun aligned poly(ε-caprolactone)/collagen nanofiber meshes on the formation of self-aligned skeletal muscle myotubes. Biomaterials.

[12] Aviss K.J., Gough J.E., Downes S. (2010). Aligned electrospun polymer fibres for skeletal muscle regeneration. Eur. Cells Mater..

[13] Jiang L., Wang L., Wang N., Gong S., Wang L., Li Q., Shen C., Turng L.S. (2018). Fabrication of polycaprolactone electrospun fibers with different hierarchical structures mimicking collagen fibrils for tissue engineering scaffolds. Appl. Surf. Sci..

[14] Teo W.E., He W., Ramakrishna S. (2006). Electrospun scaffold tailored for tissue-specific extracellular matrix. Biotechnol. J..

[15] Zhong J., Zhang H., Yan J., Gong X. (2015). Effect of nanofiber orientation of electrospun nanofibrous scaffolds on cell growth and elastin expression of muscle cells. Colloids Surf. Biointerfaces.

[16] Shimizu K., Fujita H., Nagamori E. (2009). Alignment of skeletal muscle myoblasts and myotubes using linear micropatterned surfaces ground with abrasives. Biotechnol. Bioeng..

[17] Lam M.T., Sim S., Zhu X., Takayama S. (2006). The effect of continuous wavy micropatterns on silicone substrates on the alignment of skeletal muscle myoblasts and myotubes. Biomaterials.

[18] Junkin M., Leung S.L., Whitman S., Gregorio C.C., Wong P.K. (2011). Cellular self-organization by autocatalytic alignment feedback. J. Cell Sci..

[19] Wang L., Wu Y., Guo B., Ma P.X. (2015). Nanofiber Yarn/Hydrogel Core–Shell Scaffolds Mimicking Native Skeletal Muscle Tissue for Guiding 3D Myoblast Alignment, Elongation, and Differentiation. ACS Nano.

[20] Wu Y., Wang L., Guo B., Ma P.X. (2017). Interwoven Aligned Conductive Nanofiber Yarn/Hydrogel Composite Scaffolds for Engineered 3D Cardiac Anisotropy. ACS Nano.

[21] Dong R., Ma P.X., Guo B. (2020). Conductive biomaterials for muscle tissue engineering. Biomaterials.

[22] Martins P.M., Ribeiro S., Ribeiro C., Sencadas V., Gomes A.C., Gama F.M., Lanceros-Méndez S. (2013). Effect of poling state and morphology of piezoelectric poly(vinylidene fluoride) membranes for skeletal muscle tissue engineering. RSC Adv..

[23] Shin Y.C., Lee J.H., Jin L., Kim M.J., Kim Y.J., Hyun J.K., Jung T.G., Hong S.W., Han D.W. (2015). Stimulated myoblast differentiation on graphene oxide-impregnated PLGA-collagen hybrid fibre matrices. J. Nanobiotechnol..

[24] Mckeon-Fischer K.D., Freeman J.W. (2010). Characterization of electrospun poly(L-lactide) and gold nanoparticle composite scaffolds for skeletal muscle tissue engineering. J. Tissue Eng. Regen. Med..

[25] Zhang Y., Zhang Z., Wang Y., Su Y., Chen M. (2020). 3D myotube guidance on hierarchically organized anisotropic and conductive fibers for skeletal muscle tissue engineering. Mater. Sci. Eng. C.

[26] Ahmed W.W., Wolfram T., Goldyn A.M., Bruellhoff K., Rioja B.A., Möller M., Spatz J.P., Saif T.A., Groll J., Kemkemer R. (2010). Myoblast morphology and organization on biochemically micro-patterned hydrogel coatings under cyclic mechanical strain. Biomaterials.

[27] Poinern G.E.J. (2016). Gold Nanoparticle Treated Textile-Based Materials for Potential use as Wearable Sensors. Int. J. Sci..

[28] Huang N.F., Lee R.J., Li S. (2010). Engineering of aligned skeletal muscle by micropatterning. Am. J. Transl. Res..

[29] Gunatillake P.A., Adhikari R. (2003). Biodegradable synthetic polymers for tissue engineering. Eur. Cells Mater..

[30] Chen C.N., Thompson L.D.V., Snow L.A., Placzek J.D., Boyce D.A. (2017). Chapter 1—Muscle Structure and Function. Orthopaedic Physical Therapy Secrets.

[31] Leung A.F., Hwang J.C., Cheung Y.M. (1983). Determination of myofibrillar diameter by light diffractometry. Pflügers Archiv.

[32] Park J.W., Shumaker-Parry J.S. (2014). Structural Study of Citrate Layers on Gold Nanoparticles: Role of Intermolecular Interactions in Stabilizing Nanoparticles. J. Am. Chem. Soc..

[33] Zhang H., Lin C.Y., Hollister S.J. (2009). The interaction between bone marrow stromal cells and RGD-modified three-dimensional porous polycaprolactone scaffolds. Biomaterials.

[34] Shevach M., Maoz B.M., Feiner R., Shapira A., Dvir T. (2013). Nanoengineering gold particle composite fibers for cardiac tissue engineering. J. Mater. Chem. B.

[35] Rajzer I., Rom M., Menaszek E., Fabia J., Kwiatkowski R. (2021). Conductive Polyaniline Patterns on Electrospun Polycaprolactone/Hydroxyapatite Scaffolds for Bone Tissue Engineering. Materials.

[36] Ren K., Crouzier T., Roy C., Picart C. (2008). Polyelectrolyte Multilayer Films of Controlled Stiffness Modulate Myoblast Cell Differentiation. Adv. Funct. Mater..

[37] Ogneva I.V., Lebedev D.V., Shenkman B.S. (2010). Transversal Stiffness and Young’s Modulus of Single Fibers from Rat Soleus Muscle Probed by Atomic Force Microscopy. Biophys. J..

[38] Discher D.E., Mooney D.J., Zandstra P.W. (2009). Growth factors, matrices, and forces combine and control stem cells. Science.

[39] Poveda-Reyes S., Moulisova V., Sanmartín-Masiá E., Quintanilla-Sierra L., Salmerón-Sánchez M., Ferrer G.G. (2016). Gelatin-Hyaluronic Acid Hydrogels with Tuned Stiffness to Counterbalance Cellular Forces and Promote Cell Differentiation. Macromol. Biosci..

[40] Boontheekul T., Hill E.E., Kong H.J., Mooney D.J. (2007). Regulating myoblast phenotype through controlled gel stiffness and degradation. Tissue Eng..

[41] Bazgir M., Zhang W., Zhang X., Elies J., Saeinasab M., Coates P., Youseffi M., Sefat F. (2021). Degradation and Characterisation of Electrospun Polycaprolactone (PCL) and Poly(lactic-co-glycolic acid) (PLGA) Scaffolds for Vascular Tissue Engineering. Materials.

[42] Azimi B., Nourpanah P., Rabiee M., Arbab S. (2014). Poly(ε-caprolactone)) Fiber: An Overview. J. Eng. Fibers Fabr..

[43] Browning M., Cereceres S., Luong P., Cosgriff-Hernandez E. (2014). Determination of thein vivodegradation mechanism of PEGDA hydrogels. J. Biomed. Mater. Res. Part A.

[44] Agarwal R., Blum K.M., Musgrave A., Onwuka E.A., Yi T., Reinhardt J.W., A Best C., Breuer C.K. (2019). Degradation and in vivo evaluation of polycaprolactone, poly(ε-caprolactone-co-L-lactide), and poly-L-lactic acid as scaffold sealant polymers for murine tissue-engineered vascular grafts. Regen. Med..

[45] Castellano D., Blanes M., Marco B., Cerrada I., Ruiz-Saurí A., Pelacho B., Araña M., Montero J.A., Cambra V., Prosper F. (2014). A Comparison of Electrospun Polymers Reveals Poly(3-Hydroxybutyrate)Fiber as a Superior Scaffold for Cardiac Repair. Stem Cells Dev..

[46] Rashid M., Dudhia J., Dakin S.G., Snelling S.J.B., Godoy R.D., Mouthuy P.A., Smith R.K.W., Morrey M., Carr A.J. (2020). Histopathological and immunohistochemical evaluation of cellular response to a woven and electrospun polydioxanone (PDO) and polycaprolactone (PCL) patch for tendon repair. Sci. Rep..

[47] Adewale O.B., Davids H., Cairncross L., Roux S. (2019). Toxicological Behavior of Gold Nanoparticles on Various Models: Influence of Physicochemical Properties and Other Factors. Int. J. Toxicol..

[48] Reid B., Gibson M., Singh A., Taube J., Furlong C., Murcia M., Elisseeff J. (2013). PEG hydrogel degradation and the role of the surrounding tissue environment. J. Tissue Eng. Regen. Med..

[49] Lynn A.D., Blakney A.K., Kyriakides T.R., Bryant S.J. (2011). Temporal progression of the host response to implanted poly(ethylene glycol)-based hydrogels. J. Biomed. Mater. Res. Part A.

[50] Altomare L., Gadegaard N., Visai L., Tanzi M.C., Farè S. (2010). Biodegradable microgrooved polymeric surfaces obtained by photolithography for skeletal muscle cell orientation and myotube development. Acta Biomater..

[51] Elamparithi A., Punnoose A.M., Kuruvilla S., Ravi M., Rao S., Paul S.F. (2016). Electrospun polycaprolactone matrices with tensile properties suitable for soft tissue engineering. Artif. Cells Nanomed. Biotechnol..

[52] Patz T.M., Doraiswamy A., Narayan R.J., Modi R., Chrisey D.B. (2005). Two-dimensional differential adherence and alignment of C2C12 myoblasts. Mater. Sci. Eng. B.

[53] Aubin H., Nichol J.W., Hutson C.B., Bae H., Sieminski A.L., Cropek D.M., Akhyari P., Khademhosseini A. (2010). Directed 3D cell alignment and elongation in microengineered hydrogels. Biomaterials.

[54] Hu T., Li Q., Dong H., Xiao W., Li L., Cao X. (2016). Patterning Electrospun Nanofibers via Agarose Hydrogel Stamps to Spatially Coordinate Cell Orientation in Microfluidic Device. Small.

[55] Yang K., Jung H., Lee H.R., Lee J.S., Kim S.R., Song K.Y., Cheong E., Bang J., Im S.G., Cho S.W. (2014). Multiscale, Hierarchically Patterned Topography for Directing Human Neural Stem Cells into Functional Neurons. ACS Nano.

[56] Langley B., Thomas M., Bishop A., Sharma M., Gilmour S., Kambadur R. (2002). Myostatin Inhibits Myoblast Differentiation by Down-regulating MyoD Expression. J. Biol. Chem..

[57] Hernández-Hernández J.M., García-González E.G., Brun C.E., Rudnicki M.A. (2017). The myogenic regulatory factors, determinants of muscle development, cell identity and regeneration. Semin. Cell Dev. Biol..

[58] Walsh K., Perlman H. (1997). Cell cycle exit upon myogenic differentiation. Curr. Opin. Genet. Dev..

[59] Stewart C.E.H., James P.L., Fant M.E., Rotwein P. (1996). Overexpression of insulin-like growth factor-II induces accelerated myoblast differentiation. J. Cell. Physiol..

[60] Prelle K., Wobus A.M., Krebs O., Blum W.F., Wolf E. (2000). Overexpression of Insulin-like Growth Factor-II in Mouse Embryonic Stem Cells Promotes Myogenic Differentiation. Biochem. Biophys. Res. Commun..

[61] Yang S.Y., Goldspink G. (2002). Different roles of the IGF-I Ec peptide (MGF) and mature IGF-I in myoblast proliferation and differentiation. FEBS Lett..

[62] Sacheck J.M., Ohtsuka A., McLary S.C., Goldberg A.L. (2004). IGF-I stimulates muscle growth by suppressing protein breakdown and expression of atrophy-related ubiquitin ligases, atrogin-1 and MuRF1. Am. J.-Physiol.-Endocrinol. Metab..

[63] Agarwal M., Sharma A., Kumar P., Kumar A., Bharadwaj A., Saini M., Kardon G., Mathew S.J. (2020). Myosin heavy chain-embryonic regulates skeletal muscle differentiation during mammalian development. Development.

[64] Seaborne R.A., Hughes D.C., Turner D.C., Owens D.J., Baehr L.M., Gorski P., Semenova E.A., Borisov O.V., Larin A.K., Popov D.V. (2019). UBR5 is a novel E3 ubiquitin ligase involved in skeletal muscle hypertrophy and recovery from atrophy. J. Physiol..

[65] McQuin C., Goodman A., Chernyshev V., Kamentsky L., Cimini B.A., Karhohs K.W., Doan M., Ding L., Rafelski S.M., Thirstrup D. (2018). CellProfiler 3.0: Next-generation image processing for biology. PLoS Biol..

