# Supplementary material for: Multiscale-Engineered Muscle Constructs: PEG Hydrogel Micro-Patterning on an Electrospun PCL Mat Functionalized with Gold Nanoparticles
Source: Int J Mol Sci. 2021 Dec 27;23(1):260. doi: 10.3390/ijms23010260 (PMC8745500; doi:10.3390/ijms23010260)
Supplement: Supplementary file 1 [file ijms-23-00260-s001.zip › ijms-1466511-supplementary.pdf]

Supplementary Information for Publication

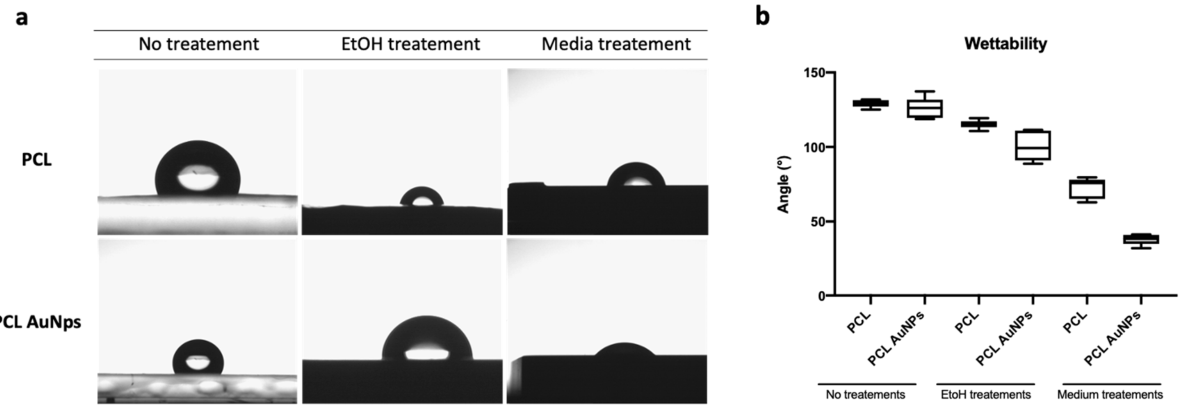

Figure S1. Wettability measurements for PCL and PCL Au NPS untreated/treated with EtOH or media by contact angle. The images (a) show the water attached to the untreated and treated substrates 1 minute after the dropping. Graph (c) gives the contact angles measured for the specimens. Data are displayed as median, quartiles, 5- and 95-percentiles (n=6, $p < 0.01$).

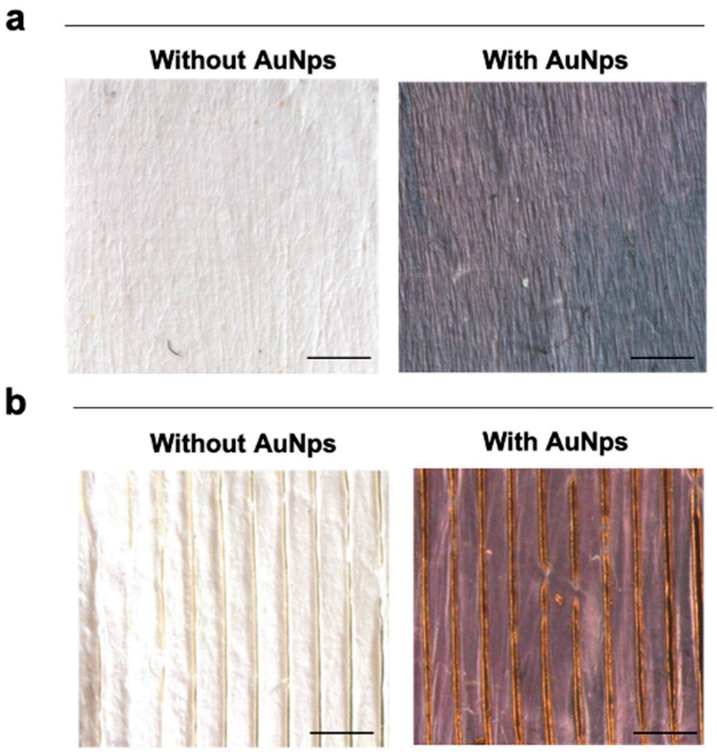

Figure S2. (a) Picture of electrospun PCL and with gold nanoparticles, (b) with micro patterned lines with a spacing of 500 μm.

Wet Scaffold	Sheet Resistance (KΩ /cm)	Conductivity (Ω $^{-1}$/cm^{-1})
PCL	49,43	2,02E-05
PCL-Au Nps	13,06	7,65E-05
PCL-Au Nps-PEG	19,96	5,00E-05

Figure S3. conductivity of the scaffolds coated obtained by using two-point probes methods.

Gene	Reference Number	Sequence (Forward/Reverse)	Product Length (bp)
RP2β (aka polr2b)	NM_153798.2	F: GATCCGTATCAGGTTCATGTCTGTA<br>R: CGGCATTCGCACCAAGGAA	123
Myogenin	NM_031189	F: CAACCCAGGAGATCATTTGCT<br>R: TGACAGACAATCTCAGTTGGG	145
MYOD	NM_010866.2	F: CATTCCAACCCACAGAAC<br>R: GGCGATAGAAGCTCCATA	125
IGF-I	NM_010512	F: CATCGTCTTTAGGAGTGTTTGTTT<br>R: TAGAATGTTATTGATAAAGGACCAAC	98
Desmin	NM_010043.2	F: ATGAGACCATCGCGGCTAAG<br>R: TTGGCTGCCTGAGTCAAGTC	82
MYH3 Emb	NM_001099635.1	F: CTACAACAGATGCGGACGGT<br>R: CAGTGCAGAGACGGTTTGGA	77

Table S1. Real-Time qPCR Primer Details.
